# Research progress on the spatiotemporal dynamics of therapy-induced senescence in remodeling the tumor microenvironment

**DOI:** 10.3389/fimmu.2026.1727142

**Published:** 2026-01-29

**Authors:** Qingqing Zhao, Yunyan Yu, Chaorui Pu, Shujuan Zheng, Lin Chen, Feng Zeng, Li Liu, Dan Li

**Affiliations:** 1 Department of General Surgery, The Second Affiliated Hospital of Zunyi Medical University, Guizhou, China; 2 Department of Laboratory Medicine, Nanchuan District People’s Hospital, Chongqing, China; 3 Department of General Surgery, Affiliated Hospital of Zunyi Medical University, Zunyi, Guizhou, China; 4 Academy of Biomedical Engineering, Kunming Medical University, Kunming, China

**Keywords:** cellular senescence, SASP, spatiotemporal dynamics, therapy-induced senescence, tumor microenvironment

## Abstract

This review systematically elaborates on the spatiotemporal dynamics and dual role of Therapy-Induced Senescence (TIS) in remodeling the Tumor Microenvironment (TME). The hallmark of TIS is the Senescence-Associated Secretory Phenotype (SASP), which drives multidimensional TME reprogramming through the secretion of various factors. These effects include the activation of Cancer-Associated Fibroblasts (CAFs), promotion of Vasculogenic Mimicry (VM), induction of metabolic reprogramming, and bidirectional regulation of the immune landscape. The article provides a focused analysis of the heterogeneous manifestations of this dual effect across different treatment stage and spatial locations, highlighting the definition of the threshold between its tumor-suppressive and tumor-promoting functions as a central current challenge. Finally, it explores future strategies involving multi-omics dynamic monitoring, artificial intelligence analysis, and spatiotemporally specific targeted interventions. In summary, this review aims to provide a theoretical foundation and translational directions for developing novel combination therapies targeting the senescent microenvironment by offering an in-depth analysis of the spatiotemporal dynamics of TIS.

## 1 Introduction and research value positioning

### 1.1 Conceptual definition and biological characteristics of therapy-induced senescence

**Figure 1:**
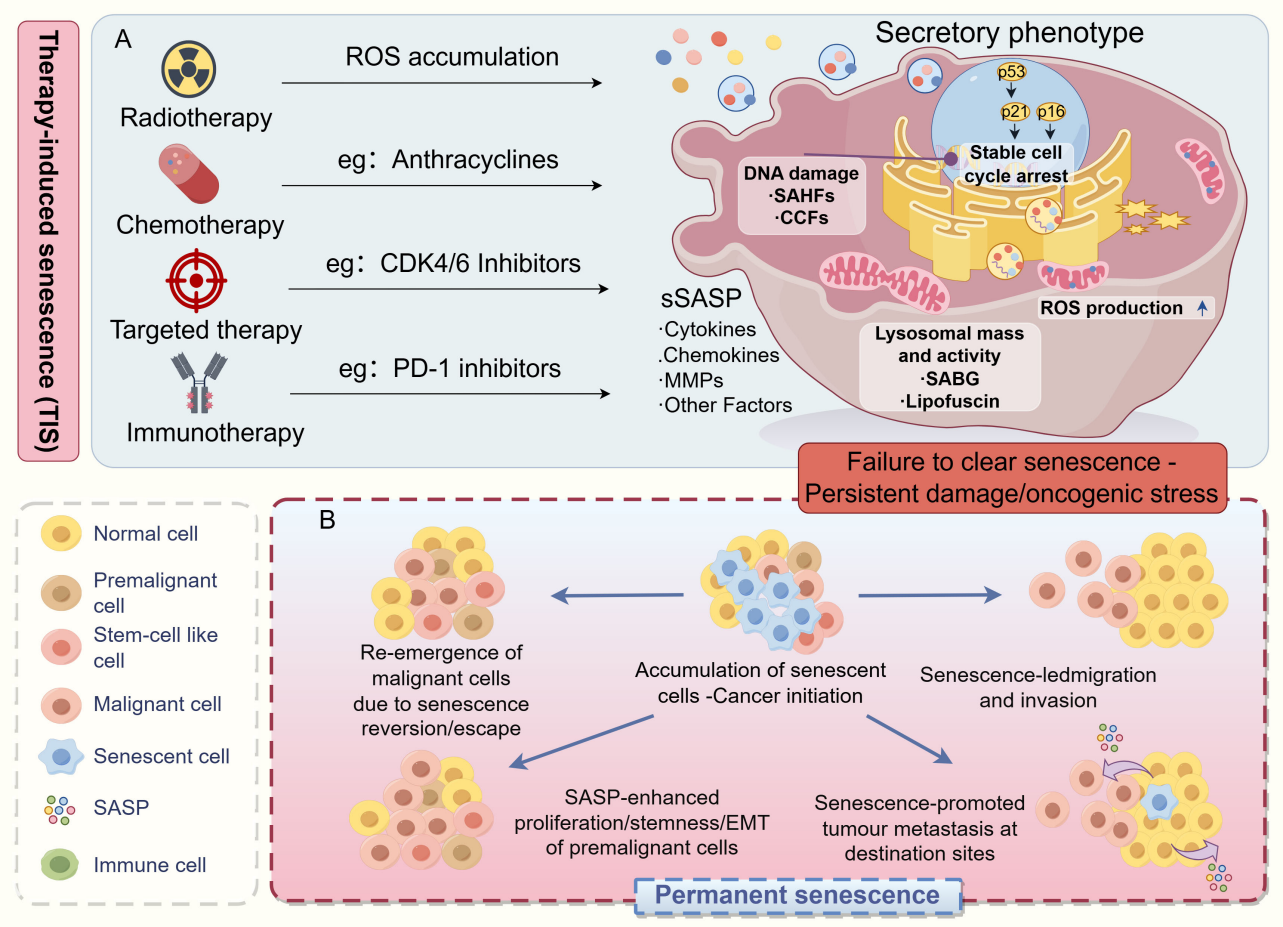
TIS promotes tumor malignant progression. **(A)** Radiotherapy, chemotherapy, targeted therapy, and immunotherapy can all trigger DNA damage, leading to senescence in both tumor and stromal cells within the TME. This results in the establishment of a stable, pro-inflammatory senescent cell population. These cells are defined by key features: enlarged, flattened morphology; permanent G1-phase cell cycle arrest; acquisition of a senescence-associated secretory phenotype (SASP) characterized by hypersecretion of inflammatory factors, growth modulators, and proteases; and extensive metabolic reprogramming. **(B)** Due to the inefficiency of the body’s senescent cell clearance mechanisms, senescent cells and their secreted SASP factors progressively accumulate within the TME. This accumulation drives the formation of a pro-tumorigenic senescent microenvironment: on one hand, senescent cells engage in bidirectional communication with surviving cancer cells via SASP factors, directly promoting their proliferation, migration, and acquisition of stem-like properties; on the other hand, active components within the SASP can increase vascular permeability, facilitating cancer cell extravasation and distant metastasis. Ultimately, the persistence of uncleared senescent cancer cells or a subpopulation of cancer cells reprogrammed to acquire stemness features collectively contributes to therapy resistance, local tumor recurrence, and distant metastasis. This figure was drawn by Figdraw.

Therapy-induced senescence (TIS) refers to a state of cellular senescence induced by anticancer treatments—including chemotherapy, radiotherapy and targeted therapy—in both tumor cells and normal cells (1–3) **Figure 1**. This particular cellular state is characterized by two core features: stable cell cycle arrest and significant morphological and physiological alterations (4). The establishment and maintenance of the senescent phenotype in TIS is driven by diverse initiating events, including persistent DNA damage response (DDR) (5), oncogenic signaling activation (6), or direct inhibition of cyclin-dependent kinases (7). These pathways ultimately converge on a redox signaling network through mechanisms such as increased mitochondrial biogenesis and reactive oxygen species (ROS) generation (8), thereby stabilizing the senescence phenotype (9). Notably, TIS cells develop a senescence-associated secretory phenotype (SASP), which remodels the surrounding microenvironment via the secretion of various cytokines, chemokines, and proteases (10). In the context of cancer therapy, TIS exhibits dual characteristics: it may restrict tumor growth through cell cycle arrest, yet it could also promote tumor progression via paracrine signaling. This duality makes TIS a critical focal point in cancer treatment research (11, 12).

### 1.2 Clinical significance of spatiotemporal dynamics in the tumor microenvironment

Studying the spatiotemporal dynamics of the tumor microenvironment (TME) is of great importance for understanding the heterogeneity of treatment responses and the patterns of tumor evolution (13, 14). The accumulation of senescent cells in the TME promotes the formation of a pro-inflammatory microenvironment and confers invasive features to tumor cells (1) **Figure 1**. Particularly in elderly patients, senescence-associated changes in the microenvironment significantly influence treatment response and clinical outcomes (5, 15). Research at single-cell resolution has revealed that TIS induces multidimensional alterations in the microenvironment, including extracellular matrix (ECM) remodeling, vascular network reorganization, and immune landscape reprogramming (16, 17). Research has revealed that in clear cell renal cell carcinoma (18) and thyroid carcinoma (19), the senescence signature within the TME is significantly correlated with remodeling of the immune microenvironment. Specifically, in patients with solid tumors receiving chemotherapy or radiotherapy (2), TIS can recruit CD8+ T cells via SASP factors such as CXCL10 (20); however, it concurrently leads to the upregulation of immune checkpoint molecules like PD-1/PD-L1 (21). In peritoneally metastatic prostate cancer, studies indicate that metastasis-specific induced senescence (non-therapy-related) drives cancer stem cell-like properties through the SASP (22). In head and neck tumors treated with conventional chemotherapy or radiotherapy (3), TIS initially manifests as cell cycle arrest but subsequently promotes tumor recurrence via ROS-mediated paracrine effects during later stages. Understanding the temporal patterns (such as differences between early and late stages of treatment) and spatial characteristics (such as heterogeneity between primary and metastatic sites) of these dynamic changes can provide a theoretical basis for developing precision treatment strategies (23, 24).

### 1.3 The dual-role effect of TIS in cancer therapy

TIS exhibits a complex dual-role effect in cancer treatment, a controversy that constitutes a key focus in current research (25). On one hand, senescence induction can halt the proliferation of tumor cells and exert tumor-suppressive effects, particularly at the precancerous stage (11). On the other hand, persistently existing senescent cells promote therapy resistance, tumor recurrence, and metastasis through SASP (9, 21). This paradoxical effect may depend on the spatiotemporal specificity of SASP components (26), the state of the immune microenvironment (27), and the efficiency of senescent cell clearance (28). It is particularly noteworthy that therapy-induced endothelial cell senescence promotes invasive behavior in tumor cells via factors such as CXCL11 (29), while the formation of a senescence-associated immunosuppressive microenvironment limits the long-term efficacy of immunotherapy (21, 30). These findings highlight the crucial value of in-depth analysis of the spatiotemporal dynamics of TIS in overcoming current therapeutic bottlenecks (8, 21).

## 2 Research progress on the core molecular mechanism of TIS

### 2.1 Key players in the senescence-associated secretory phenotype

The most prominent feature of TIS is the activation of the SASP, a complex secretome composed of multiple pro-inflammatory factors (31). Key SASP components include cytokines and chemokines (interleukin (IL)-6, IL-8), growth factors (VEGF, GM-CSF, TGF-β) and matrix metalloproteinases (MMPs) (5, 14). These secreted factors remodel the TME through paracrine signaling, which may enhance anti-tumor immune responses but also promote immunosuppression and tumor progression (32, 33).

SASP secretion is not a one-time event but a dynamically regulated process under precise hierarchical control. Its transcriptional regulation relies on a complex signaling cascade: in the initial phase, sustained DDR signals activate the p38 MAPK pathway via ATM/ATR–Chk1/2 (34, 35), and cooperate with the transcription factor AP-1 (c-Fos/c-Jun) to initiate the first wave of pro-inflammatory factors (IL-6, IL-8) (36, 37). In the mid-to-late phases, the NF-κB signaling pathway is strongly activated (through ROS or GATA4 accumulation resulting from autophagy inhibition) and serves as the primary driver of SASP, inducing extensive expression of chemokines (CXCL1, CXCL10), proteases (MMPs), and growth factors (TGF-β, amphiregulin) (38–40). In addition to these classical pathways, the cytosolic DNA sensor cGAS-STING pathway has emerged as a central regulator of the inflammatory SASP, particularly in the context of therapy-induced DNA damage (41). Furthermore, epigenetic reprogramming (H3K36me2/3 modifications) and mRNA stability regulation (loss of ZFP36L1) further shape and sustain specific SASP profiles, enhancing its complexity and sustainability. It is noteworthy that SASP composition varies significantly across tumor types and treatment regimens, and this heterogeneity directly influences the ultimate biological effects of TIS (42, 43).

### 2.2 DNA damage repair pathways and epigenetic regulation

The DDR serves as a core molecular mechanism triggering TIS. Conventional therapies such as chemotherapy and radiotherapy induce persistent DNA damage, activating the ATM/ATR–Chk1/2–p53/p21 signaling axis and leading to permanent cell cycle arrest (5, 16). This mechanism is consistently observed across cancer types. For instance, analyses of breast cancer patient samples post-therapy reveal a significant upregulation of senescence markers alongside DDR activation and SASP expression (44). Furthermore, models of prostate and lung cancer have demonstrated that DNA-damaging agents directly induce the expression of these senescence hallmarks (45).

Epigenetic remodeling acts as a critical switch determining cell fate toward senescence (46–48). Characteristic changes in histone modifications include a reduction in repressive marks (H3K27me3) and an increase in activating marks (H3K4me3, H3K36me3) at pro-senescence gene loci such as the p16INK4A locus (49, 50). Furthermore, HIRA-mediated deposition of histone variant H3.3 (51) and nuclear lamina disruption accompanied by loss of heterochromatin—due to degradation of Lamin B1, promoting senescence-associated heterochromatin foci formation (52)—collectively establish an open chromatin state that sustains the expression of senescence-associated genes (53, 54).

These epigenetic alterations not only lock in cell cycle exit but also directly regulate SASP, making senescence a stable and difficult-to-reverse program. For instance, chemotherapy promotes tumor cell senescence by suppressing the SLX4 complex-mediated DNA repair pathway, a process involving cCCT2 protein and SUMO conjugation that leads to compromised DNA repair function (20). Concurrently, radiotherapy induces not only direct DNA damage but also significant epigenetic reprogramming (e.g., histone modifications, DNA methylation), which further influences cellular radiosensitivity and contributes to the establishment of the senescence phenotype (55).

### 2.3 The cGAS-STING pathway: a double-edged sword in cancer therapy

The cGAS-STING pathway, as a cytosolic DNA sensor (56), serves as a core hub regulating the inflammatory SASP and plays a key role in therapy-induced DDR (57, 58). Genotoxic stress, the primary effector mechanism of cancer therapies such as chemotherapy and radiotherapy, leads to the abnormal accumulation of nuclear DNA and mitochondrial DNA (mtDNA) fragments in the cytoplasm (59). These DNA fragments are recognized by cyclic GMP-AMP synthase, which catalyzes the production of the second messenger 2’3’-cGAMP. This subsequently activates STING, which triggers the TBK1/IKK kinase cascade to induce the activation of transcription factors IRF3 and NF-κB (60, 61). This pathway not only drives the production of type I interferons (e.g., IFN-β) but also regulates the secretion of various classic SASP factors, including IL-6 and TNF-α, thereby shaping a complex inflammatory milieu (58, 62). In cancer therapy, activation of the cGAS-STING pathway exhibits a dual regulatory “double-edged sword” nature. In terms of antitumor effects, radiotherapy can activate the cGAS-STING pathway by inducing DNA damage, promoting the recruitment of tumor-infiltrating CD8^+^ T cells. For instance, in hepatocellular carcinoma models, host STING deficiency impairs immune surveillance functions (63, 64). In homologous recombination-deficient high-grade serous ovarian cancer, cGAS-STING-mediated limited SASP can improve the efficacy of immune checkpoint inhibitors (65). Regarding pro-tumor effects, mitochondrial dysfunction can lead to mtDNA leakage through channels formed by VDAC1 oligomerization, persistently activating the cGAS-STING pathway and promoting the secretion of SASP with pro-tumorigenic effects (59). In colorectal cancer cells, mitochondrial stress caused by serine deficiency induces type I IFN secretion through the mtDNA-cGAS-STING1 axis, accelerating the senescence phenotype (66).

Notably, tumor cells can suppress cGAS-STING pathway activity through metabolic reprogramming, such as enhanced glycolysis (67). Conversely, the use of nanomaterials to deliver radiosensitizers like hafnium oxide can synergistically enhance this pathway, offering potential to overcome the dose limitations of radiotherapy (68). Additionally, regulatory factors such as thioredoxin reductase 1 (TXNRD1) interact with cGAS, which can enhance its enzymatic activity to promote immune surveillance or potentially contribute to the pro-tumorigenic effects of senescent cells (69). These findings provide a molecular basis for the “double-edged sword” nature of the cGAS-STING pathway in cancer therapy and offer important theoretical support for developing temporal regulation strategies, such as controlled release of agonists using nanocarriers (70, 71).

### 2.4 Evolutionarily conserved senescence signaling networks

Evolutionarily conserved senescence signaling networks regulate TIS across species and tissues. The p16INK4a/Rb and p53/p21 pathways represent two highly conserved axes governing senescence. P16 activation has been demonstrated to mediate TIS following chemotherapy or radiotherapy, thereby modulating tumor progression and DDR signaling (72, 73). Metabolic reprogramming serves as another conserved hallmark of senescence. Pyruvate dehydrogenase kinase 4 (PDK4) promotes both DNA damage levels and SASP secretion by enhancing glycolysis, while inhibition of PDK4 alleviates senescence-associated phenotypes (74).

In addition to glycolytic reprogramming, increasing evidence indicates that alterations in lipid metabolism play a key role in establishing and maintaining the senescent phenotype (75, 76). Senescent cells exhibit significant disturbances in lipid metabolism, characterized by enhanced lipid uptake, massive accumulation of lipid droplets, and abnormal deposition of cholesterol in lysosomes (77, 78). This metabolic remodeling has dual biological effects: on one hand, it contributes to senescence initiation by triggering lipotoxic stress (79); on the other hand, it provides substrates for β-oxidation to meet the increased energy demands of senescent cells, supporting their long-term survival (11, 80). Notably, cellular sensitivity to TIS varies across tissue types. For example, cancer-associated fibroblasts (CAFs) within the TME undergo TIS, acquiring a pro-tumorigenic SASP that promotes cancer cell invasion and drug resistance (81). These conserved mechanisms offer potential molecular targets for developing broad-spectrum anti-senescence intervention strategies (6, 82).

## 3 Spatiotemporal remodeling of the tumor microenvironment by TIS

### 3.1 Stromal remodeling: temporal activation of CAFs and spatial reorganization of the ECM

**Figure 2:**
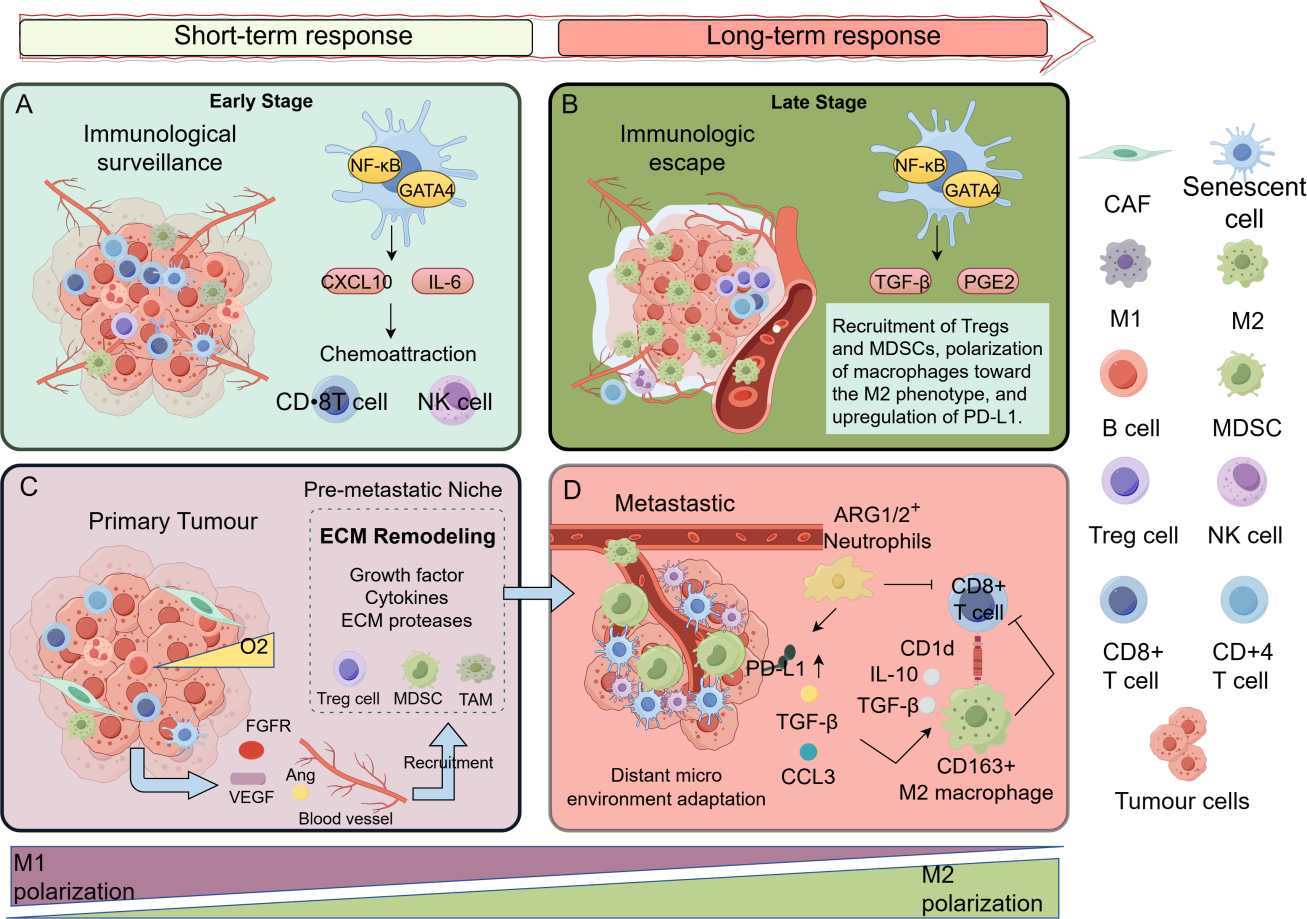
Spatiotemporal evolution of the therapy-induced senescent microenvironment. **(A)** Early-stage therapy: Following initial treatment, tumors rapidly develop a large number of therapy-induced senescent cells, accompanied by significant infiltration of immune cells. The microenvironment exhibits an immunoactivated state, possessing the potential to suppress tumor growth. **(B)** Late-stage therapy: With continued treatment, TISnt cells accumulate persistently within the primary focus, and immunosuppressive cells gradually become dominant. The microenvironment reverses from an immunoactivated to a deeply immunosuppressive state, creating favorable conditions for tumor recurrence. **(C)** Primary tumor: The primary tumor enters a senescent state post-treatment, forming a pre-metastatic niche. This microenvironment is under immune surveillance, and senescent cells initiate adaptive reprogramming, laying the groundwork for subsequent explosive metastasis. **(D)** Metastatic focus: Within the metastatic focus, tumor cells that successfully escape senescence or cancer cell subclusters that acquire stemness proliferate extensively and recruit a large number of immunosuppressive cells, constructing an immune-excluded niche. This ultimately leads to the explosive growth of clinically apparent metastatic tumors. This figure was drawn by Figdraw.

TIS significantly remodels the tumor stroma by triggering the activation of CAFs, a process characterized by dynamic temporal features and spatial heterogeneity **Figure 2**. Studies have shown that TIS can induce stromal fibroblasts to enter a senescent state and acquire a pro-tumorigenic CAF-like phenotype (1, 11). The function of these senescent CAFs evolves over time through the secretion of SASP factors (2, 83): they may be involved in tissue repair responses in the early stages (84), but persistently present senescent CAFs exhibit strong pro-tumorigenic properties through ECM remodeling (85, 86) and the secretion of pro-inflammatory factors (1). For instance, in pancreatic ductal adenocarcinoma (PDAC), senescent CAFs activate pro-survival signaling pathways in tumor cells via paracrine SASP factors (87). Furthermore, these senescent CAFs exhibit unique metabolic reprogramming, characterized by a shift from oxidative phosphorylation to aerobic glycolysis (9), supporting tumor growth through mechanisms such as lactate secretion. A notable example is found in prostate cancer, where lactate secreted by senescent CAFs can specifically modulate the transcriptional programs of tumor cells through epigenetic mechanisms, such as histone lactylation (88, 89).

In the spatial dimension, the interaction between TIS and stromal cells shows significant heterogeneity. By secreting factors such as TIMP1 (90, 91), senescent CAFs form a pro-invasive ECM structure. This ECM remodeling has been demonstrated to enhance tumor invasiveness and therapy resistance (92, 93). Single-cell analyses reveal that in the invasive front regions of tumors, senescent cells and CAFs form a unique spatial interaction network, mediating stromal remodeling through the integrin-FAK-ERK-Akt1 signaling axis and ECM-degrading enzymes (84). In contrast, the tumor core is dominated by interactions between senescent tumor cells and immune cells (26, 94). This spatial heterogeneity directly leads to differential treatment responses in distinct areas of the tumor (12).

### 3.2 Angiogenesis and vasculogenic mimicry: spatiotemporal homeostatic mechanisms

TIS releases a complex network of angiogenic modulators through the SASP (95, 96). In the early stages, SASP may promote vascular normalization via factors like VEGFA (97); however, in later stages, SASP shifts towards a pro-inflammatory phenotype, driving pathological vascular remodeling (39, 98). Notably, a synergistic effect exists between TIS and vasculogenic mimicry (VM) formation in hypoxic microenvironments (99, 100). This phenomenon, where tumor cells directly form vascular networks (101, 102), is markedly different from traditional endothelial-dependent angiogenesis (103).

Senescent cells remodel the TME through paracrine mechanisms: 1) The PI3K/Akt pathway influences VM formation by regulating EphA2 phosphorylation (104); 2) TIS specifically upregulates pro-tumorigenic factors like IL-6, CXCL8, and TGF-β (10, 95), and these core SASP components show a significant positive correlation with VM markers (105, 106); 3) In metastatic sites, VM, together with vascular co-option, forms a dual escape mechanism contributing to therapy resistance (100, 103). This may explain the limitations of anti-angiogenic therapies in advanced patients (107). VM formation is closely associated with high tumor malignancy and poor prognosis (99), while the dynamic evolution of SASP (108) suggests the need for spatiotemporal regulation strategies: targeting pro-vascular normalization factors in the early phase and inhibiting pathological SASP components in the later phase (3). Simultaneously, combination therapies targeting key VM-forming pathways (such as EphA2/PI3K) may help overcome resistance to traditional anti-angiogenic treatments (106).

### 3.3 Metabolic reprogramming: nutrient competition and metabolite exchange

Metabolic reprogramming, as a core mechanism by which TIS reshapes the TME, dynamically regulates TME evolution through metabolic crosstalk among multiple cell types. Studies have shown that senescent breast cancer cells, by secreting extracellular vesicles, inhibit the mTOR signaling pathway in CAFs, triggering metabolic reprogramming in CAFs, which in turn enhances the release of pro-inflammatory SASP factors and promotes stromal remodeling (1, 6). In colorectal cancer models, TIS-induced CAFs exhibit significant lipid metabolism reprogramming. The metabolites they secrete, such as ketone bodies and lactate, can activate invasion-related pathways in tumor cells via epigenetic modifications, thereby accelerating metastasis (5).

This metabolic interaction displays marked spatial heterogeneity. For example, in the lung cancer microenvironment, tumor cells predominantly rely on mitochondrial oxidative phosphorylation for energy, whereas CAFs enhance the glycolytic pathway to secrete metabolic substrates like pyruvate and glutamine (109, 110). This bidirectional metabolic coupling not only maintains energy homeostasis but, more importantly, participates in the regulation of immune evasion through metabolite exchange (111).

Notably, TIS systemically remodels the TME via SASP factors: on one hand, it induces glycolytic dysfunction in T cells (112, 113); on the other, it promotes lipid accumulation in myeloid-derived suppressor cells (MDSC) (5, 6). This dynamic metabolic reprogramming ultimately leads to the formation of an immunosuppressive microenvironment (109). Recent studies further reveal that metabolic competition within the TME exhibits dynamic evolution. During the early stages of treatment, TIS cells alter local nutrient distribution by secreting SASP factors (such as IL-6, TGF-β) (10, 114). As treatment progresses, metabolite exchange between senescent cells and immune cells further intensifies immune suppression (112). This spatiotemporal dynamic nature suggests that timely interventions targeting metabolic crosstalk may emerge as novel therapeutic strategies (110).

### 3.4 Immune microenvironment: the dual roles in spatiotemporal evolution

The remodeling of the immune microenvironment by TIS is a dynamically evolving process, with its dualistic nature manifesting at different time windows and spatial locations **Figure 2**. In the early stages, the SASP promotes the chemotaxis and migration of CD8^+^ T cells by releasing various cytokines (20), while pro-inflammatory factors like IL-6 can activate NK cell and T cell immunity (115), theoretically establishing a microenvironment conducive to immune surveillance. However, in an immunosuppressive tumor immune microenvironment or during later stages, the SASP composition dynamically shifts to become dominated by immunosuppressive factors like TGF-β (4, 116), leading to immune evasion.

From a spatial perspective, senescent cells can establish localized immune-privileged niches by forming physical barriers or secreting specific factors (such as immunosuppressive factors mediated by CAFs and macrophages) (117, 118), resulting in the exclusion or functional suppression of effector immune cells. Single-cell studies confirm significant heterogeneity among TIS cells, with different subsets exerting opposing effects on immune regulation (116). In PDAC, activated pancreatic stellate cells and heterogeneous CAF subsets remodel the TME (119, 120), forming a complex interaction network with immune cells that collectively promotes immunosuppression and therapy resistance (121, 122). Notably, the immunomodulatory effects of SASP are highly context-dependent: under specific conditions (such as in combination with EZH2 inhibitors), SASP can reactivate the secretion of pro-inflammatory factors (CCL2, CXCL9/10) (115), enhancing NK cell and T cell immunity; whereas in an immunosuppressive microenvironment, the same SASP may exacerbate immune tolerance (4, 116). This bidirectional regulatory characteristic suggests that therapeutic strategies targeting TIS must carefully consider the cell types undergoing senescence, the SASP composition profile, and their spatiotemporal dynamics (123).

In addition to SASP-mediated effects, TIS can also enhance immune recognition through SASP-independent mechanisms. Studies have shown that senescent cells upregulate interferon signaling pathways and mechanisms associated with MHC class I molecules (124, 125), and present senescence-associated self-peptides that effectively activate CD8+ T cells (126). Particularly in the context of cancer, senescent tumor cells significantly enhance the expression of antigen presentation machinery (APM) components, promoting the assembly of MHC class I complexes (127). This upregulation can even be further potentiated by IFNγ treatment, thereby markedly increasing the visibility of senescent cells to cytotoxic T cells.

## 4 integrated spatiotemporal dynamics and therapeutic implications

Current evidence indicates that the impact of TIS on the TME exhibits dynamic evolutionary characteristics, and its biological effects are governed by precise spatiotemporal regulatory mechanisms (1, 3, 114). This process typically begins with an initial phase of therapeutic benefit, characterized by tumor growth inhibition and immune activation. Over time, however, it gradually transitions into a chronic pro-tumorigenic phenotype, ultimately leading to the formation of an immunosuppressive microenvironment (10, 11). This temporal evolution pattern reveals a critical therapeutic intervention window (74, 128).

Notably, the spatiotemporal heterogeneity of TIS is also reflected in functional differences at different anatomical sites. Senescent cells in the primary tumor may remain under immune surveillance, whereas those in metastatic niches tend to drive tumor regrowth within an immune-privileged microenvironment (129–131). This spatial specificity necessitates that treatment strategies be personalized according to the anatomical location of the lesions (90, 92). A deeper understanding of the interplay between the temporal dynamics of SASP components and the spatial context of the tumor is crucial for overcoming current therapeutic bottlenecks (5, 25, 132). Among the core scientific challenges is defining the dynamic threshold at which TIS transitions from tumor-suppressive to tumor-promoting effects—a threshold that itself varies with treatment duration and the spatial location of the lesion (4, 9). Recent advances in cutting-edge technologies such as spatial transcriptomics and multiplex immunofluorescence have provided unprecedented direct evidence for deciphering the spatial distribution of TIS and its role in mediating intercellular communication.

Spatial transcriptomics enables the analysis of transcriptomic features while preserving the spatial information of tissues *in situ*, thereby revealing the spatial organization patterns of different cell types within the TME (133, 134). For instance, in a study on angiosarcoma, this technology not only delineated the topological features of “immune-hot” and “immune-cold” regions but also identified localized immune clusters even within immune-cold tumors (135). By integrating single-cell sequencing data, spatial transcriptomics can further construct high-resolution cell interaction networks. For example, in a hepatoblastoma study, the combination of single-cell/single-nucleus RNA sequencing with spatial transcriptomic analysis systematically unveiled the spatial architecture of tumor-associated fibrosis and its key signaling pathways (136, 137). Multiplex immunofluorescence technology supports *in situ* detection with multiple markers, enabling the quantification of spatial interactions between tumor and immune cells. For instance, a colorectal cancer study systematically analyzed 1,269 multiplex fluorescence images and developed a computational model to evaluate multi-level spatial interaction patterns within the TME (138). This technique also effectively complements spatial transcriptomics. For example, in an intrahepatic cholangiocarcinoma study, the co-localization of MARCO^+^ tumor-associated macrophages (TAMs) and CTSE^+^ tumor cells, initially identified by spatial transcriptomics, was subsequently validated in 20 samples using multiplex immunofluorescence (139). However, the application of these powerful spatial technologies to specifically map and characterize the distribution of senescent cells within the TME—termed the TIS spatial pattern—remains notably underexplored. While the aforementioned studies provide a robust methodological framework for dissecting tissue architecture, a dedicated investigation into the spatial niches, heterogeneity, and interaction networks of therapy-induced senescent cells is currently lacking. This gap is critical because the functional impact of therapy-induced senescent cells is heavily context-dependent and influenced by their precise localization and neighboring cell types. Consequently, to fully realize the potential of senescence-targeting therapies, it is imperative to first elucidate the spatial architecture of TIS.

Taken together, the emerging capabilities of spatial technologies and the identified knowledge gap highlight that future therapeutic paradigms must move beyond static models and develop dynamic targeting strategies capable of responding in real time to the spatiotemporal changes in the senescent microenvironment (140–142).

## 5 Progress in clinical translation

### 5.1 Development of TIS biomarkers and treatment response prediction

The development of biomarkers for TIS faces major challenges. Current research primarily focuses on DDR markers and components of the SASP **Table 1**. Analyses of clinical samples suggest that cell-cycle inhibitory proteins such as p16INK4a and p21WAF1/CIP1 may serve as potential TIS biomarkers, though their expression in human tumor tissues shows significant heterogeneity (140). Recent studies using single-cell sequencing have identified specific upregulation of chemokines such as CXCL11 in endothelial cells following treatment, suggesting their potential as novel biomarkers for predicting TIS-related adverse outcomes (143). Notably, the emergence of a senescence-like secretory signature in stromal cells induced by PARP inhibitor therapy has been significantly associated with clinical treatment failure (144). These findings highlight that the development of TIS biomarkers must account for responses in both tumor cells and microenvironmental components.

**Table 1:** Potential biomarkers for TIS detection and monitoring: preclinical evidence and research directions.

Category	Candidate biomarker	Potential clinical utility	Key challenges	Refs
Cell Cycle Inhibitors	p16INK4a, p21CIP1/WAF1, p53	p16: High specificity for senescence but variable sensitivity; strong correlation with poor prognosis in some cancers. p21: Often transient; may indicate early senescence but lacks long-term specificity.	Tissue heterogeneity; expression in both tumor and stromal cells.	(140, 145–148)
SASP Factors	IL-6, IL-8, MCP-1, TGF-β1	Elevated post-chemotherapy/radiotherapy; dynamic changes may predict outcome. Liquid biopsy potential. However, low specificity.	Lack of cancer- and therapy-specific SASP signatures; high background noise.	(10, 46, 95, 149, 150)
DNA Damage Response	CHK2, γ-H2AX, Lamin-B1, p53 Ser15 phosphorylation, PAI-1, MBNL2	Direct marker of therapy-induced DNA damage, the primary trigger of TIS. High sensitivity for recent genotoxic insult.	Short-lived; does not specifically mark stable senescence.	(140, 151–155)
Senescence-Associated β-Galactosidase	Lysosomal β-galactosidase activity at pH 6.0	The most widely used histological marker. High specificity in controlled conditions.	Not applicable to liquid biopsy. Sensitivity affected by tissue fixation and can be present in non-senescent confluent cells.	(156, 157)
Epigenetic Modification	H3K4me3, H2BK58	Stable and heritable marks that lock in the senescent state.	Technically challenging and costly to assay in clinical samples. Clinical utility is still exploratory.	(49, 158)

### 5.2 Synergistic mechanisms of combination immunotherapy

The synergy between TIS and immunotherapy exhibits a “double-edged sword” characteristic. On one hand, SASP-derived factors such as IL-6 and TGF-β can promote the recruitment of MDSCs, fostering an immunosuppressive microenvironment (21). On the other hand, TIS induced by certain specific chemotherapy regimens enhances antigen presentation and activates CD8^+^ T cells (159). Emerging strategies are now designed to actively steer this pro-inflammatory potential of TIS. For instance, in pancreatic cancer models, the combination of a RAS(ON) inhibitor and a CDK4/6 inhibitor potently induce tumor cell senescence, remodeling the TME to include tertiary lymphoid structure (TLS)-like aggregates. The subsequent addition of a CD40 agonist then robustly engages this primed immune environment, leading to CD4^+^ T cell and IFN-γ-dependent durable tumor control, effectively establishing a state of long-term tumor-immune equilibrium (160). Preclinical studies have shown that radiation-induced senescent tumor cells promote T cell infiltration through the CCL5–CXCR3 axis, and when combined with PD-1 inhibitors, significantly improve treatment efficacy (161). This concept—that removing senescent cells can rejuvenate immunity—finds support beyond cancer models. A key illustration is the use of senolytic chimeric antigen receptor (CAR) T-cells targeting the senescence-associated surface marker uPAR. Although this strategy was demonstrated in aged mice, where it reversed the accumulation of exhausted T-cell subsets and rejuvenated mucosal immune function (162), it establishes a critical precedent: targeted clearance of senescent cells can directly alleviate immunosuppression. This fundamental principle lends strong rationale to applying senolytic strategies against the persistent, immunosuppressive TIS cells in cancer to enhance immunotherapy. However, persistently surviving TIS cells may induce T cell exhaustion via PD-L1 upregulation (92), explaining the limited efficacy of combination therapy observed in some clinical trials. To circumvent this resistance, novel delivery systems have been developed to precisely target senescent cells. For example, galacto-oligosaccharide-coated nanoparticles (GalNP) encapsulating cytotoxic drugs (e.g., doxorubicin) are specifically cleaved by the high lysosomal β-galactosidase (SABG) activity in senescent cells, leading to targeted drug release. This strategy enhances the efficacy of senolytics while minimizing systemic toxicity, offering a promising approach to eliminate the persistent, immune-suppressive TIS cell population without harming healthy tissue (163). Currently, predictive models for immunotherapy response based on SASP component analysis are under development, particularly focusing on spatiotemporally specific immune regulatory mechanisms in solid tumors such as head and neck cancer (3). The integration of single-cell analyses is further refining our understanding, revealing that ТIS is not a monolithic state but comprises distinct cell subpopulations with varying immunoregulatory potentials (26, 164). This heterogeneity underscores the necessity for personalized combination strategies that either exploit the immunostimulatory capacity of certain TIS states or precisely eliminate the immunosuppressive ones to achieve optimal synergy with immunotherapy (3, 160).

### 5.3 Validation of senolytic agents in preclinical models

The sequential therapeutic strategy of “induce and eliminate” has shown promise in models such as prostate cancer, wherein chemotherapy is first used to induce TIS, followed by senolytic agents to clear senescent cells (165). Preclinical studies have confirmed that the combination of dasatinib and quercetin effectively eliminates radiation-induced senescent endothelial cells and inhibits their pro-metastatic effects on tumor cells (143). A core challenge in clinical translation is the heterogeneity of senescent cells and the diversity of their dependency networks (166, 167). Senescent cells generated by different cell types and induction methods exhibit vast differences in anti-apoptotic pathways (168, 169). For instance, some depend on BCL-2/BCL-xL and are sensitive to navitoclax, while others may rely heavily on PI3K/AKT, HSP90, or EGFR signaling for survival (144, 170–172). Therefore, the future direction is not to seek a “universal” senolytic, but to develop biomarker-guided precision senolytic strategies. Examples include detecting senescent cell surface markers (uPAR, DPP4, ICAM-1) or specific SASP profiles to direct the use of corresponding antibody-drug conjugates or bispecific antibodies for targeted clearance (173). Additionally, developing combination therapies targeting senescent cell anti-apoptotic pathways—such as BCL-2 inhibitors combined with PI3Kδ inhibitors—may help overcome mono-agent resistance and broaden the spectrum of efficacy (174). Recent advances include the design of dual inhibitors targeting mTOR, a core regulator of SASP, which can maintain the anti-proliferative effect of TIS while suppressing its tumor-promoting secretory functions (26). This strategy of inhibiting pro-tumorigenic SASP aligns with the emerging concept of ‘senomorphics’ or ‘senostatics’ – a class of therapeutic agents designed to modulate the deleterious phenotypes of senescent cells without directly inducing their apoptosis (131, 175). This approach is conceptually distinct from “senolytics,” which aim to clear senescent cells (176). The use of mTOR inhibitors, along with inhibitors targeting other key SASP regulatory pathways such as NF-κB or p38 MAPK, represents a promising senomorphic strategy (96, 177). Studies have shown that small molecules like SR9009 can suppress both the SASP and the DDR by activating the NRF2 pathway, while concurrently reducing ROS levels through the modulation of antioxidant enzyme expression (96). Similarly, drugs such as ruxolitinib significantly reduce SASP factors and senescence markers, while also mitigating mitochondria-mediated apoptosis (178). These findings provide a theoretical foundation for developing spatiotemporally specific combination therapies targeting TIS.

## 6 Current challenges and controversies

### 6.1 Spatiotemporal regulation of SASP composition

As a core effector mechanism of TIS, the SASP exhibits significant temporal and spatial heterogeneity. Studies have shown that SASP comprises various pro-inflammatory factors, chemokines, and growth factors whose dynamic changes in the TME directly affect treatment outcomes (128). However, the precise regulatory mechanisms controlling SASP remain unclear, particularly given the substantial differences in SASP expression profiles across treatment stages and tumor sites (161). Furthermore, newly identified regulatory mechanisms such as the NOTCH signaling pathway suggest that SASP may be tissue-specific (161), posing considerable challenges to the development of broad-spectrum modulation strategies. Single-cell studies also reveal marked heterogeneity in SASP signatures even among senescent cells within the same tumor region (26), further complicating targeted intervention.

### 6.2 Defining the transition threshold between pro-tumor and anti-tumor effects

TIS exhibits a typical “double-edged sword” effect in cancer therapy, and defining the balance between its pro-tumor and anti-tumor functions remains a critical challenge for clinical translation. On one hand, SASP can enhance treatment efficacy by activating anti-tumor immunity (128); on the other hand, the same secretory phenotype may promote tumor progression and therapy resistance (179). This paradoxical effect appears closely linked to concentration-dependent thresholds of SASP components (42), yet quantitative standards to define such thresholds are still lacking. Responses to SASP also vary significantly across cancer types—for instance, a SASP scoring system established in glioblastoma (180) may not be applicable to other malignancies. Additionally, factors such as treatment timing and duration influence the net biological outcome (11), introducing substantial uncertainty into clinical decision-making.

### 6.3 Research limitations due to heterogeneity in clinical samples

Spatiotemporal heterogeneity in the TME poses major obstacles in TIS research. Clinical sample analyses indicate that primary and metastatic lesions respond differently to TIS (22), and such spatial variability limits the generalizability of findings. The relationship between genomic features (single nucleotide variants, SNVs) and microenvironmental dynamics remains poorly understood (181), hindering deeper mechanistic insight into TIS heterogeneity. Technologically, current methods such as spatial transcriptomics still lack sufficient resolution to fully capture dynamic processes (182). Furthermore, interpatient variability in treatment responses (21) and the complex interactions among diverse cell types in the TME (CAFs, TAMs) (81, 183) add to the difficulty of clinical translation. Together, these factors contribute to a significant gap between laboratory discoveries and clinical applications.

## 7 Future research directions

**Figure 3:**
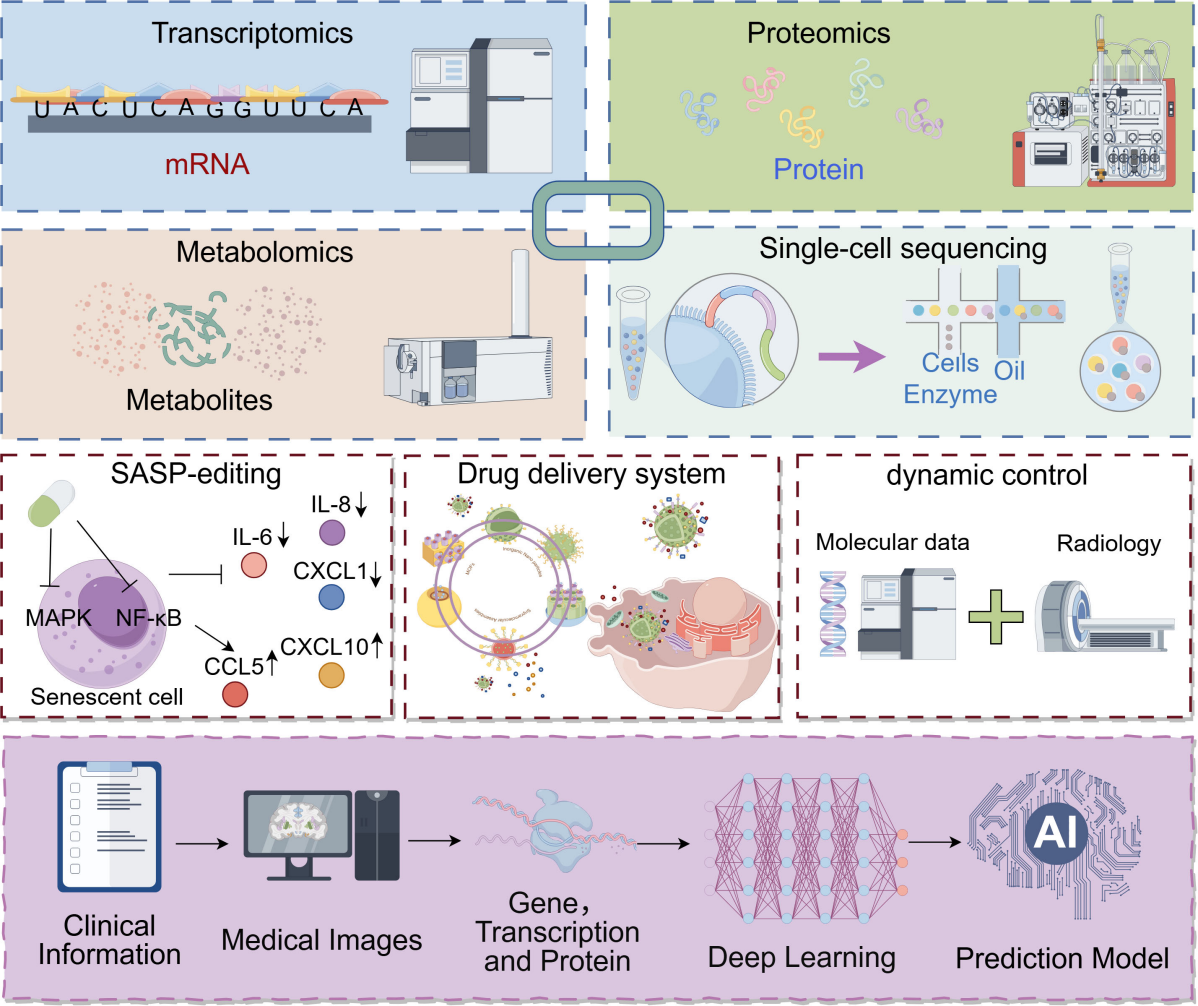
Future precision therapeutic strategies targeting the senescent microenvironment. Multi-omics dynamic monitoring: Integrates technologies such as single-cell sequencing, spatial transcriptomics, and proteomics to achieve high-resolution dynamic monitoring of the TME during cancer treatment. Precision intervention and dynamic assessment: Involves the development of small-molecule inhibitors to selectively neutralize or clear key pro-tumorigenic SASP factors, and the design of smart drug delivery systems responsive to specific biomarkers. These approaches are combined with imaging and liquid biopsy techniques to track therapy-induced senescent cells in real-time. AI-assisted decision-making: Employs artificial intelligence models to integrate multi-omics and clinical data, enabling precise identification of senescent cell subtypes, assessment of their pro-tumorigenic risk, and prediction of their evolutionary trajectory. This provides a scientific basis for timely and personalized intervention. This figure was drawn by Figdraw.

Building on the current evidence, we propose several speculative yet promising avenues for future investigation, which are crucial for translating the biology of TIS into clinical applications **Figure 3**.

### 7.1 Dynamic monitoring technologies integrating multi-omics data

There is an urgent need to develop dynamic monitoring platforms capable of integrating multi-omics data—such as transcriptomics, proteomics, and metabolomics—to decipher real-time changes in the TME during TIS (179). In particular, high temporal-resolution detection methods are required to track dynamic changes in SASP composition and capture critical transition points in microenvironmental remodeling during early and late treatment phases (26). The application of single-cell multi-omics technologies will help uncover heterogeneous interaction networks between TIS cells and immune cells, potentially offering new insights into the mechanisms underlying the shift between pro-tumor and anti-tumor effects (184).

### 7.2 Spatiotemporally specific targeted intervention strategies

Building on recent advances, a potentially breakthrough approach moving beyond conventional senolytic strategies could involve the development of spatiotemporally specific reprogramming technologies. This strategy might encompass three innovative dimensions: 1. Precision SASP Editing (SASP-editing): By targeting key signaling pathways such as NF-κB or p38 MAPK using small molecule inhibitors or epigenetic drugs (39), it might be possible to selectively reshape the SASP. This approach would aim to suppress the secretion of tumor-promoting factors while enhancing immunostimulatory cytokine expression—all without eliminating senescent cells. Epigenetic reprogramming has been shown to dynamically modulate the SASP expression profile, suggesting it as a more nuanced strategy that may better preserves tissue homeostasis compared to outright cell clearance (185). 2. Smart Drug Delivery Systems: A promising direction is to develop stimuli-responsive nanoparticles or liposomes that target specific microenvironmental signals to accurately deliver senolytics or SASP-modifying drugs to tumor areas enriched with senescent cells, thereby potentially avoiding off-target effects on healthy tissues. 3. Dynamic Intervention Timing: After chemotherapy/radiotherapy, monitoring TIS dynamics via imaging or liquid biopsy (detecting specific SASP factors) may help identify a potential therapeutic window. Based on the kinetics of SASP secretion and immune microenvironment remodeling, it is hypothesized that intervention before the peak of pro-tumor SASP secretion or at the initial stage of immunosuppressive microenvironment formation could achieve maximum therapeutic efficacy. Through spatiotemporal regulation of ROS signaling and DNA damage repair pathways, selective inhibition of pro-tumor SASP components may be achieved while preserving their immune-activating functions (186). Precise intervention in the dynamic balance between pro-angiogenic and anti-angiogenic signals could represent a strategy to break the vicious cycle of TIS-mediated therapy resistance (29). In addition, region-specific targeted delivery systems would likely need to be designed to address microenvironmental differences between primary and metastatic sites (187).

## 8 Summary and key conclusions

TIS represents a critical yet complex biological response during cancer treatment, characterized by spatiotemporal dynamics and a dual-role nature. This review synthesizes recent advances regarding the impact of TIS on the TME, leading to the following core conclusions: TIS mediates multifaceted regulatory effects through the SASP, wherein secreted cytokines, chemokines, and proteases can either activate antitumor immune surveillance or foster an immunosuppressive microenvironment (10, 188). This functional duality is closely tied to the spatiotemporal specificity of SASP components, generally exerting tumor-suppressive effects early in treatment while potentially driving recurrence at later stages (19, 129). The remodeling of the TME by TIS occurs across multiple dimensions: it promotes tumor invasion via CAF activation and ECM remodeling (92); disrupts the dynamic balance between pro- and anti-angiogenic signals (29); induces metabolic reprogramming through nutrient competition and metabolite exchange (144); and exerts bidirectional immunomodulation by simultaneously activating and suppressing immune responses (25, 188). Single-cell analyses further reveal substantial heterogeneity in these remodeling processes between primary and metastatic tumors (187). Key challenges in clinical translation include the difficulty in defining the threshold between pro-tumor and anti-tumor effects due to dynamic SASP changes (25), the role of senescent endothelial cells in promoting invasion via factors like CXCL11 (29), and the influence of age-related senescent microenvironments on treatment response in elderly patients (12, 15). Although combining senolytics with immunotherapy demonstrates synergistic potential, clinical applicability remains limited by sample heterogeneity (26, 189). Future research should prioritize developing multi-omics-integrated dynamic monitoring platforms to resolve TIS spatiotemporal evolution (26), designing precision interventions targeting specific senescent subpopulations (28, 189), and establishing organoid models and AI-driven predictive systems to address translatability gaps related to age and heterogeneity (12). A deeper mechanistic and dynamic understanding of TIS will provide a critical foundation for novel senescence-targeting combination therapies (5).

## Glossary

AP-1: Activator Protein-1
Ang: Angiopoietin
APM: Antigen Presentation Machinery
ATM: Ataxia Telangiectasia Mutated
ATR: Ataxia Telangiectasia and Rad3-Related
CAFs: Cancer-Associated Fibroblasts
CAR: Chimeric Antigen Receptor
cGAS: cyclic GMP-AMP Synthase
CCFs: cytoplasmic chromatin fragments
Chk1/2: Checkpoint Kinase 1/2
DDR: DNA Damage Response
ECM: Extracellular Matrix
FGFR: Fibroblast Growth Factor Receptor
GATA4: GATA Binding Protein 4
GM-CSF: Granulocyte-Macrophage Colony-Stimulating Factor
IFN: Interferon
IL: Interleukin
MAPK: Mitogen-Activated Protein Kinase
MDSC: myeloid-derived suppressor cell
MMPs: Matrix Metalloproteinases
NK Cell: Natural Killer Cell
PD-1: Programmed Cell Death Protein 1
PDAC: Pancreatic Ductal Adenocarcinoma
PDK4: Pyruvate Dehydrogenase Kinase 4
PD-L1: Programmed Cell Death Ligand 1
PGE2: Prostaglandin E2
ROS: Reactive Oxygen Species
SABG: Senescence-Associated Beta-Galactosidase
SASP: Senescence-Associated Secretory Phenotype
sSASP: secreted Senescence-Associated Secretory Phenotype
SAHFs: Senescence-Associated Heterochromatin Foci
SCAPs: Senescent Cell Anti-apoptotic Pathways
STING: Stimulator of Interferon Genes
TAM: tumor-associated macrophage
TIS: Therapy-Induced Senescence
TLS: Tertiary Lymphoid Structure
TME: Tumor Microenvironment
TNF: Tumor Necrosis Factor
VEGF: Vascular Endothelial Growth Factor
VM: Vasculogenic Mimicry
TGF-β: Transforming Growth Factor Beta
TXNRD1: thioredoxin reductase 1.
